# Plasma kynurenines and prognosis in patients with heart failure

**DOI:** 10.1371/journal.pone.0227365

**Published:** 2020-01-10

**Authors:** Anders Lund, Jan Erik Nordrehaug, Grete Slettom, Stein-Erik Hafstad Solvang, Eva Kristine Ringdal Pedersen, Øivind Midttun, Arve Ulvik, Per Magne Ueland, Ottar Nygård, Lasse Melvaer Giil

**Affiliations:** 1 Department of Clinical Science, University of Bergen, Bergen, Norway; 2 Department of Cardiology, Stavanger University Hospital, Stavanger, Norway; 3 Department of Heart Disease, Haukeland University Hospital, Bergen, Norway; 4 Department of Internal Medicine, Haraldsplass Deaconess Hospital, Bergen, Norway; 5 Bevital AS, Bergen, Norway; 6 Laboratory of Clinical Biochemistry, Haukeland University Hospital, Bergen, Norway; Macquarie University, AUSTRALIA

## Abstract

**Background:**

Metabolites of the kynurenine pathway (mKP) relate to important aspects of heart failure pathophysiology, such as inflammation, energy-homeostasis, apoptosis, and oxidative stress. We aimed to investigate whether mKP predict mortality in patients with heart failure.

**Methods:**

The study included 202 patients with heart failure (73.8% with coronary artery disease (CAD)), propensity score matched to 384 controls without heart disease, and 807 controls with CAD (71%). All underwent coronary angiography and ventriculography at baseline. Plasma mKP, pyridoxal 5’phosphate (PLP) and CRP were measured at baseline. Case-control differences were assessed by logistic regression and survival by Cox regression, adjusted for age, gender, smoking, diabetes, ejection fraction, PLP, eGFR and CRP. Effect measures are reported per standard deviation increments.

**Results:**

Higher plasma levels of kynurenine, 3- hydroxykynurenine (HK), quinolinic acid (QA), the kynurenine-tryptophan-ratio (KTR) and the ratio of HK to xanthurenic acid (HK/XA) were detected in heart failure compared to both control groups. The mortality rate per 1000 person-years was 55.5 in patients with heart failure, 14.6 in controls without heart disease and 22.2 in CAD controls. QA [HR 1.80, p = 0.013], HK [HR 1.77, p = 0.005], HK/XA [HR 1.67, p < 0.001] and KTR [HR 1.55, p = 0.009] were associated with increased mortality in patients with heart failure, while XA [HR 0.68–0.80, p = 0.013–0.037] were associated with lower mortality in all groups. HK and HK/XA had weak associations with increased mortality in CAD-controls.

**Conclusion:**

Elevated plasma levels of mKP and metabolite ratios are associated with increased mortality, independent of CAD, in patients with heart failure.

## Introduction

Heart failure is associated with chronic inflammation, oxidative stress and cell-death, reflected by an increase in relevant biomarkers [1–3]. Recruitment and activation of monocytes in the myocardium is considered a key pathophysiological process. Activated monocytes and macrophages, stimulate myocardial fibrosis [4] and generate metabolites of the kynurenine pathway (mKP) from the essential amino acid tryptophan. mKP may mediate immunomodulation, oxidant defence and apoptosis [5], which are considered pathogenic features in the development of heart failure.

**Fig 1:**
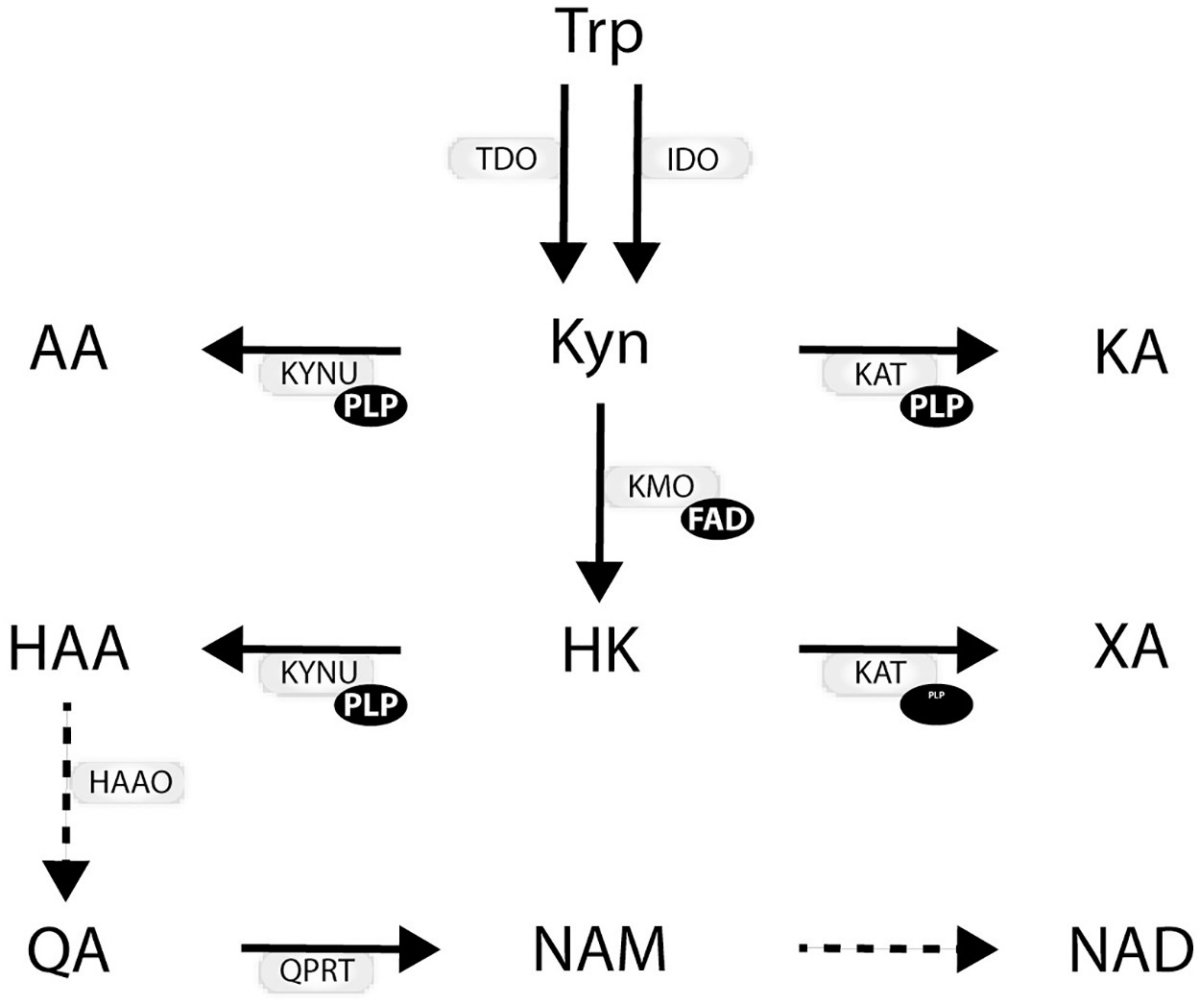
The kynurenine pathway. The kynurenine pathway (KP) is the major route of Trp degradation. The first step is the formation of formylkynurenine from Trp, catalysed by hepatic tryptophan 2,3-dioxygenase (TDO), predominantly expressed in the liver, or indoleamine-2,3-dioxygenase (IDO), expressed in monocytes. Formylkynurenine is rapidly converted to kynurenine (Kyn). Kyn in turn is converted to a variety of metabolites, many of which have immunomodulatory effects. These include 3-hydroxykynurenine (HK, formed by the flavin adenine dinucleotide (FAD)-dependent kynurenine monooxygenase (KMO)), kynurenic acid (KA) and xanthurenic acid (XA) (products of the pyridoxal 5-phosphate (PLP)-dependent kynurenine aminotransferase (KAT)), anthranilic acid (AA) and 3-hydroxyanthranilic acid (HAA) (products of the pyridoxal 5-phosphate (PLP)-dependent kynureninase (KYNU). HAA is further fully oxidized and in the process produce picolinic acid (not shown in figure) or converted to quinolinic acid (QA) in several steps and further to nicotinic acid mononucleotide and ultimately to NAD+. Abbreviations: KP, kynurenine pathway; AA, anthranilic acid; FAD, flavin adenine dinucleotide; HAA, 3-hydroxyanthranilic acid; HAAO, 3-hydroxyanthranilate 3,4-dioxygenase; HK, 3-hydroxykynurenine; IDO, indoleamine (2,3)-dioxygenase; KA, kynurenic acid; KAT, kynurenine aminotransferase; KMO, kynurenine 3-monooxygenase; Kyn, kynurenine; KYNU, kynureninase; PLP, pyridoxal 5´-phosphate; TDO, tryptophan (2,3)-dioxygenase; Trp, tryptophan; QA, quinolinic acid; QPRT, quinolinate phosphoribosyltransferase; XA, xanthurenic acid; NAM, nicotinamide; NAD, nicotinamide adenine dinucleotide.

Fig 1 illustrates the kynurenine pathway (KP). Glucocorticoids and cytokines induce the rate-limiting enzymes tryptophan 2,3-dioxygenase (TDO) and indoleamine 2,3-dioxygenase (IDO) who converts tryptophan (Trp) to kynurenine (Kyn) [6]. Interferon gamma (IFN-γ) activates the enzymes IDO and KMO [5]. Consequently, an increase in IFN-γ activity in patients with heart failure [7], may potentially result in elevated circulating levels of mKP.

Plasma levels of mKP predict prognosis in both healthy populations and in patients with cardiovascular disease. In a community-based cohort [8], mKP levels predicted all-cause mortality and cardiovascular mortality. In patients with stable angina pectoris and coronary artery disease (CAD), mKP predicted incident myocardial infarction [9]. The levels of the metabolite Kyn were increased in heart failure compared to controls [10]. In the same study, Kyn was associated with increased mortality, but other mKP were not measured [10]. CAD is frequent in patients with heart failure, and mKP have been hypothesized as a driver of CAD itself [8].

In this study, we aimed to investigate if Trp and mKP predict all-cause mortality in patients with established heart failure, compared to 2 control groups without heart failure, but with or without CAD.

## Methods

### Study participants

Patients were recruited from the Western Norway Coronary Angiography Cohort (WECAC) between 1999–2004. All patients (N = 4164) underwent elective coronary angiography at Haukeland- or Stavanger University Hospital mostly due to chest pain. Heart failure was identified in 202 patients. Two control groups were matched to these patients (as described below).

Patient characteristics were obtained at baseline. This included a medical history, self-administered questionnaires, a review of hospital records, and a clinical examination. A trained cardiologist performed cardiac catheterization. Significant coronary stenoses were confirmed in orthogonal views. The number of significantly stenosed vessels defined the severity of CAD (0 to 3). A stenosis in the left main stem counted as a two-vessel disease. Left ventricular ejection fraction (EF) was determined by ventriculography.

Self-report, a fasting glucose ≥ 7.0 mmol/L or non-fasting glucose > 11.1 mmol/L defined diabetes. Self-report and a serum cotinine level ≥ 85 nmol/L defined smoking status [11]. Blood samples were drawn at baseline. All-cause mortality was registered from The Norwegian Cause of Death Registry, which covers 98% of the Norwegian population [12]. The censoring date in this study, 31.12.2013, was the time of the last link between the study and the registry.

### Case definitions and propensity score matching

Heart failure was diagnosed in patients with symptoms, or on treatment, with a left ventricular ejection fraction (LVEF) ≤40% or LVEF <50% and New York Heart Association (NYHA) class ≥2, resulting in 202 patients with heart failure. One-hundred and forty-one (69.8%) patients reported a diagnosis of heart failure. Renin-angiotensin inhibitors were used by 155 (76.7%), beta-blockers by 147 (72.8%), loop diuretics by 121 (59.9%) and 187 (92.5%) used either. Exclusion criteria were severe pulmonary disease, known cancer at baseline, dialysis-demanding renal failure and other terminal illness.

**Fig 2:**
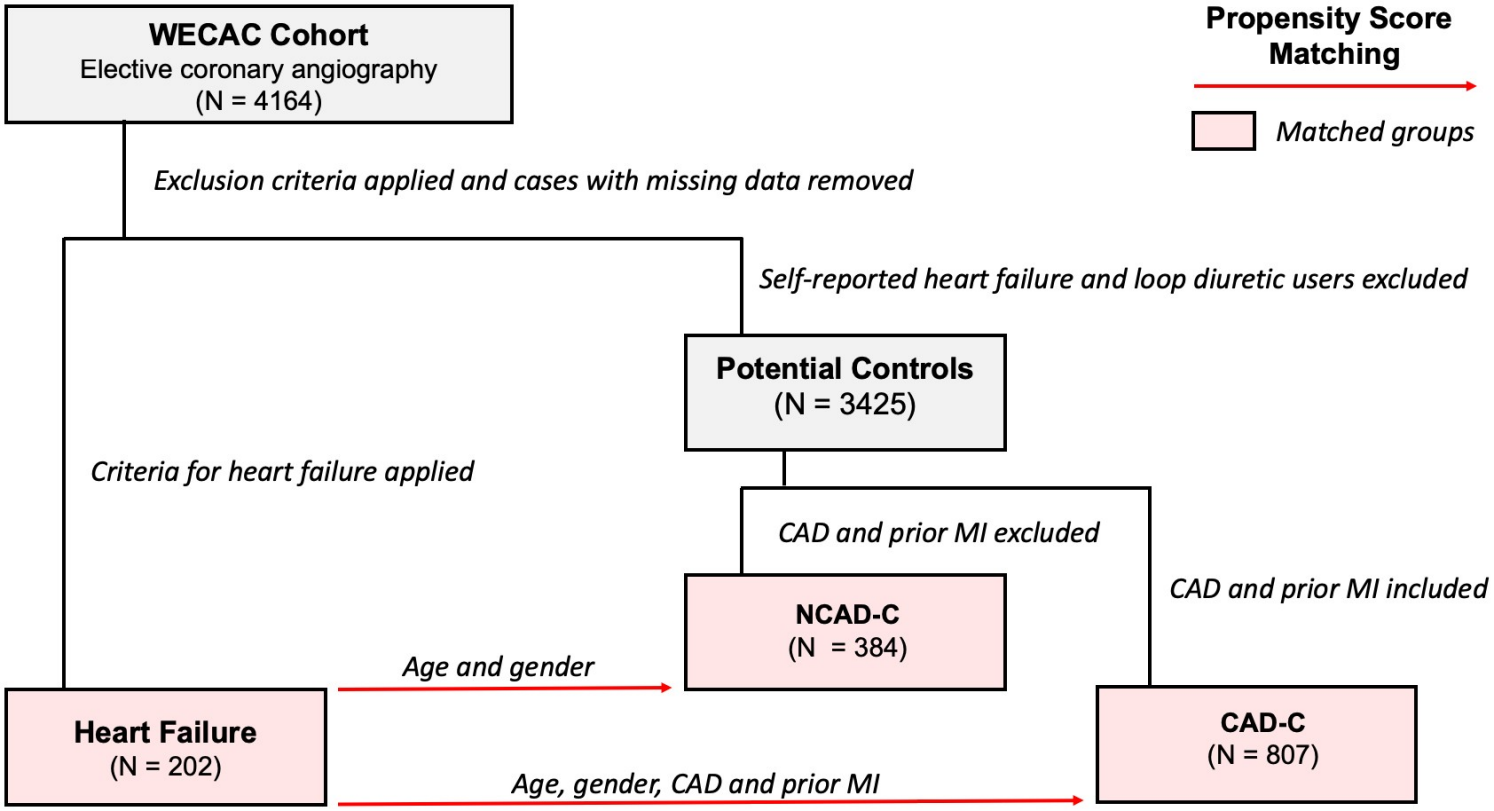
Propensity score matching of cases and controls. 3425 patients were identified as potential controls, who did not meet criteria for heart failure and did not report having a diagnosis of heart failure and did not use loop diuretics. These potential controls were propensity score matched to the heart failure group based on age, gender, number of vessels affected by CAD and prior MI generating controls with similar levels of CAD as the heart failure group (CAD-C). A subgroup of the potential controls without CAD and prior MI were propensity score matched based on age and gender, generating a control group without CAD (NCAD-C). Abbreviations: CAD, coronary artery disease represented by number of vessels affected by significant coronary artery stenosis on coronary angiography; MI, myocardial infarction; WECAC, western norway coronary angiography cohort; CAD-C coronary artery disease controls; NCAD-C, non-coronary artery disease controls.

Two control groups were generated by three steps. First, patients from WECAC without criteria for heart failure, self-reported heart failure, and who were not on loop diuretics were considered potential controls. Second, cases with missing data (< 5%) were removed. This left 3425 potential controls. From these potential controls one subgroup was generated without CAD (NCAD-C), based on no significant stenoses on coronary angiography and no previous myocardial infarction. All potential controls were matched to the heart failure group on age, gender, the severity of CAD, and/or previous myocardial infarction using propensity score matching, generating a control group with proportional CAD to the HF group (CAD-C). The subgroup without CAD was matched to the heart failure group on age and gender, again using propensity score matching, generating CAD-free controls (NCAD-C). Fig 2 illustrates a flow-chart of the matching process.

Propensity score matching was performed using the nearest neighbor algorithm, with random matching order and caliper set to 0.2, as recommended [13]. Cardiovascular risk factors and renal function were not matched for, as this would generate selection bias. Due to the limited number of matching variables, the groups were treated as independent in subsequent statistical analyses.

### Ethics

This study was approved by the Regional Ethics Committee (REC) West, University of Bergen, Faculty of Medicine, Bergen, Norway, with identification number 2013/2022. All patients provided written consent after the study procedures had been explained in detail.

### Measurements of metabolites and biomarkers

Blood samples were collected in tubes containing ethylenediamine tetraacetic acid (EDTA) from non-fasting participants. Trp, Kyn, 3-hydroxykynurenine (HK), kynurenic acid (KA), anthranilic acid (AA), 3-hydroxyanthranilic acid (HAA), xanthurenic acid (XA), quinolinic acid (QA), and PLP were measured by Bevital AS (http://www.bevital.no) using liquid chromatography-tandem mass spectrometry (LC-MS/MS) assay [14]. The Kyn to Trp ratio (KTR) was calculated as Kyn (in nmol/L) divided by Trp (in μmol/L), as was the ratio of HK to XA (HK/XA).

Serum C-reactive protein (CRP) was measured using an ultrasensitive immunoassay by the Behring nephelometer II system (N Latex CRP mono, Behring Diagnostics, Marburg, Germany). Creatinine was measured by the hospital laboratories. Estimated glomerular filtration rate (eGFR) was calculated using the Chronic Kidney Disease Epidemiology Collaboration (CKD-EPI) formula.

### Missing data

Fifty-four patients did not have measurements of QA in the heart failure group. These missing data could be predicted from the independent variables (*X*^2^ 20.3, p = 0.009, Little’s test). The missing data for QA were imputed by multiple imputation. The strongest independent predictors of QA, Kyn, and HK were used for imputation together with age, gender, current smoking (estimated from cotinine [11]), eGFR, EF, PLP, CRP, and diabetes. The missing data were imputed 200 times and pooled estimates for all imputations are reported for QA [15].

### Statistics

T-tests, Mann Whitney U-test, Chi-square, and log-rank tests were used for univariate analysis. Variables with non-normal distributions were transformed prior to multivariate analyses, according to Tukey’s ladder of powers (square root, logarithms, 1/square root, and inverse). All variables were subsequently standardized to their respective z-scores, calculated as z = (x– μ) / σ, where x is the variable, μ its mean and σ its standard deviation. Each resulting data-point is then scaled to reflect the number of standard deviations it lies from a mean of zero. The aim of standardization was to make effect sizes of variables on different scales and transformations more comparable.

Metabolites were analyzed in separate multivariate models to avoid collinearity. We used logistic regression to estimate the differences between cases and controls, adjusted for potential confounders (CRP, diabetes, eGFR, PLP and Trp). From the significant models in logistic regression, we wanted to evaluate if kynurenines could be used as diagnostic biomarkers of heart failure. We evaluated metabolites and derived ratios separately. Using Lasso with all significant metabolites identified, or ratios, we identified the optimal multivariate logistic regression model using the default cross-validation method to identify λ. From this model, the predicted probabilities from the logistic regressions were used as the predictor of the diagnostic categories using receiver operating characteristic (ROC) analysis, which were presented graphically. The area under the curve (AUC) was estimated. We also estimated sensitivity and specificity directly from the logistic regression models using the post-hoc analysis tool “estat class” in Stata 16.

The hazard for all-cause mortality associated with metabolite levels was estimated using Cox proportional hazard analysis for patients with heart failure and the two control groups (CAD-C and NCAD-C). These analyses were adjusted for age, gender, CRP, diabetes, current smoking, eGFR, PLP, and Trp. The assumption of proportional hazards was checked in stratified analyses for categorical variables. For continuous variables, it was evaluated by inspecting Shoenfeld residuals and by performing the Therneau and Grampsch test.

The Benjamini-Hochberg procedure was used to adjust for multiple comparisons, at a false discovery rate (FDR) of 0.05 and FDR-adjusted p-values are reported (Q-values). The number of tests were estimated by summarizing all case-control comparisons and the hypotheses of association with mortality for the heart failure group. Statistical analyses and corresponding graphs were conducted with Stata 15 and 16 (Stata Statistical Software: Release 15 and 16. College Station, TX: StataCorp LLC), R version 3.3.2 (packages: PM Match and p.adjust).

### Confounders

The potential confounders eGFR, Trp, diabetes, PLP and CRP were selected *a priori* and included in the multivariate models [16], as the primary aim was to identify independent associations with mKP and end points. Both renal function and protein intake could be reduced in heart failure [17, 18]. Plasma levels of mKP are strongly related to renal function [19, 20], and availability of the substrate Trp is linked to protein intake and downstream levels [21]. Trp was not included as a confounder when KTR was evaluated, as KTR is a product of Trp and Kyn. Patients with diabetes have higher levels of mKP and diabetes is an individual predictor for heart failure [22, 23]. PLP is an essential co-factor for KAT and KYNU [20], and have been found to be related to plasma levels of several mKP [24]. Elevated CRP is associated with a diagnosis of heart failure and increased mortality in heart failure [25]. CRP is an unspecific marker of systemic inflammation and could thus be indirectly associated with IDO-activation [26], supported by association with plasma levels of several mKP [27].

## Results

### Characteristics of the study participants

Table 1 summarizes group characteristics. Compared with both control groups, patients with heart failure had lower eGFR, higher CRP, and a higher prevalence of diabetes. PLP levels were lower in patients with heart failure compared to the NCAD-C group. The heart failure and both control groups were well matched for age and gender. The CAD-C group was, in addition, well matched on previous MI and number of vessels with coronary artery stenosis. Among the patients with open coronary arteries at baseline, there were higher prevalence of previous myocardial infarction and/or percutaneous coronary intervention in the CAD-C group compared to patients with heart failure.

**Table 1:** Baseline characteristics and group mortality.

Clinical Characteristics	Heart failure (N = 202)	Controls (N = 1191)	
		NCAD-C (N = 384)	CAD-C (N = 807)
Age, years; mean (SD) ^a^	63.1 (9.1)	61.5 (9.1)	62.8 (9.8)
Male; N (%) ^b^	155 (76.7)	277 (72.1)	606 (75.1)
Body mass index; mean (SD) ^a^	26.1 (4.2)	26.3 (3.7)	26.6 (3.8)
**Risk Factors and Possible Confounders**			
Diabetes; N (%) ^b^	32 (16.3)	21 (5.5)*	79 (9.8)*
Current smoker; N (%) ^b^	63 (31.2)	101 (26.3)	260 (32.2)
Hypertension; N (%) ^b^	97 (48.7)	169 (44.0)	402 (49.8)
eGFR, ml/min/1.73^2^; mean (SD) ^a^	83 (28)	88.9 (14)**	88.4 (15.1)**
CRP, mg/L; median (IQR) ^c^	2.9 (3.6)	1.5 (2.4)**	1.9 (3.2)**
PLP, nmol/L; median (IQR) ^c^	43.4 (38.7)	47.0 (34.4)*	40.8 (31.4)
**Cardiac Disease**			
Prior myocardial infarction; N (%) ^b^	135 (66.8)	0 (0)**	520 (64.4)
Coronary artery stenosis; N (%) ^b^			
0	53 (26.2)^#^	384 (100)**	234 (29)^$^
1	30 (14.9)	0**	119 (14.8)
2	31 (15.3)	0**	108 (13.4)
3	88 (43.6)	0**	346 (42.9)
Ejection fraction; median (IQR) ^c^	34.0 (8.4)	70 (5)**	65 (10)**
**All-Cause Mortality**			
Deceased, N (%) ^d^	94 (46.5)	56 (14.6)**	180 (22.3)**
Mortality rate, per 1000 PY	55.5	14.6	22.2
Mean follow up time, years	9.6	10.0	10.0

Abbreviations: CAD-C, controls with coronary artery disease; CRP, C-reactive protein; N, number of patients; NCAD-C, controls free of coronary artery disease; PY, person-years; PLP, pyridoxal 5’phosphate; SD, standard deviation.

Tests comparing heart failure to controls:

^a^ Students T-test

^b^ Chi square test

^c^ Mann-Whitney U test

^d^ Log-rank test

^#^ 9 (17%) with previous MI. 3 (6.7%) with previous PCI. 43 (81%) with no previous MI or PCI.

^$^ 83 (35.5%) with previous MI. 50 (21%) with previous PCI. 139 (59.4%) with no previous MI or PCI.

* p-value < 0.05,

** p-value < 0.001

### Metabolites of the kynurenine pathway in patients with heart failure and control groups

Unadjusted plasma levels of Kyn, HK, KA, AA, HAA, QA, KTR, and HK/XA were higher in patients with heart failure compared to both control groups (S1 Table). Adjusted for confounders and multiple comparisons, patients with heart failure had higher levels of Kyn, HK, QA, KTR, and HK/XA ratio compared to both control groups (Table 2). From the metabolites, we identified a model with Trp and Kyn (and HK in CAD-C group) and both KTR and HK/XA, using Lasso. ROC curves from multivariate logistic regression gave an AUC of 0.7 for Trp and Kyn and an AUC of 0.69 for KTR and HK/XA with HF versus NCAD-C as outcome, and an AUC of 0.69 for Trp, Kyn and HK, and an AUC of 0.65 with HF versus CAD-C as outcome. Further, adding these kynurenines to models with confounders have only minimal improvement in AUC (See S1 and S2 Figs in supporting material).

**Table 2:** Tryptophan and metabolites of the kynurenine pathway in heart failure. Adjusted analyses ^a^.

	HF versus NCAD-C ^b^				HF versus CAD-C ^c^			
	OR	95% CI	p	Q ^d^	OR	95% CI	p	Q ^d^
Trp	1.08	0.89–1.31	.44	.53	1.22	1.04–1.45	.017*	.034*
Kyn	1.94	1.47–2.56	< .001**	< .001**	1.69	1.35–2.11	< .001**	< .001**
HK	1.50	1.13–2.00	.005*	.014*	1.46	1.16–1.85	.001*	.005*
KA	1.15	0.90–1.48	.26	.36	1.11	0.90–1.37	.31	.40
XA	0.90	0.72–1.14	.40	.50	0.96	0.79–1.17	.71	.71
AA	1.16	0.94–1.45	.16	.25	1.05	0.87–1.26	.62	.64
HAA	1.07	0.87–1.33	.52	.59	0.89	0.74–1.08	.23	.35
QA	1.54	1.18–2.01	.002*	.007*	1.44	1.15–1.79	.001*	.005*
KTR	1.72	1.31–2.26	< .001**	.001*	1.41	1.14–1.74	.001*	.005*
HK/XA	1.30	1.04–1.64	.024*	.045*	1.42	1.16–1.73	.001*	.005*

Case-control differences evaluated by logistic regression. Abbreviations: AA, anthranilic acid; CI, confidence interval; HAA, 3-hydroxyanthranilic acid; HK, 3-hydroxykynurenine; HK/XA, 3-hydroxykynurenine-xanthurenic acid ratio; KA, kynurenic acid; KTR, kynurenine-tryptophan ratio; Kyn, kynurenine; OR, odds ratio; SD; standard deviation; QA, quinolinic acid; XA, xanthurenic acid.

^a^ Odds ratios per 1 SD of the predictor by logistic regression.

^b^ Heart failure (HF, N = 202) vs controls without coronary artery disease (NCAD-C, N = 384) as outcome, adjusted for diabetes, glomerular filtration rate, pyridoxal 5’phosphate, C-reactive protein and Trp (not Trp in KTR model).

^c^ HF (N = 202) vs controls with coronary artery disease (CAD-C, N = 807). Covariates as in model ^b^.

^d^ p-value adjusted for multiple comparisons in the study (Benjamini-Hochberg; 0.05)

* p-value (p) or q-value (Q) < 0.05,

** p-value or q-value < 0.001

### Metabolites of the kynurenine pathway and all-cause mortality

Mortality differed significantly between the heart failure (46.5% died), NCAD-C (14.6%) and CAD-C (22.3%) groups. The median follow-up time was similar (9.6 years in heart failure, 10 years in NCAD-C and CAD-C). The mortality rate per 1000 person-years was 55.5 in patients with heart failure, 14.6 in CAD controls and 22.2 in NCAD controls (Table 1).

**Fig 3:**
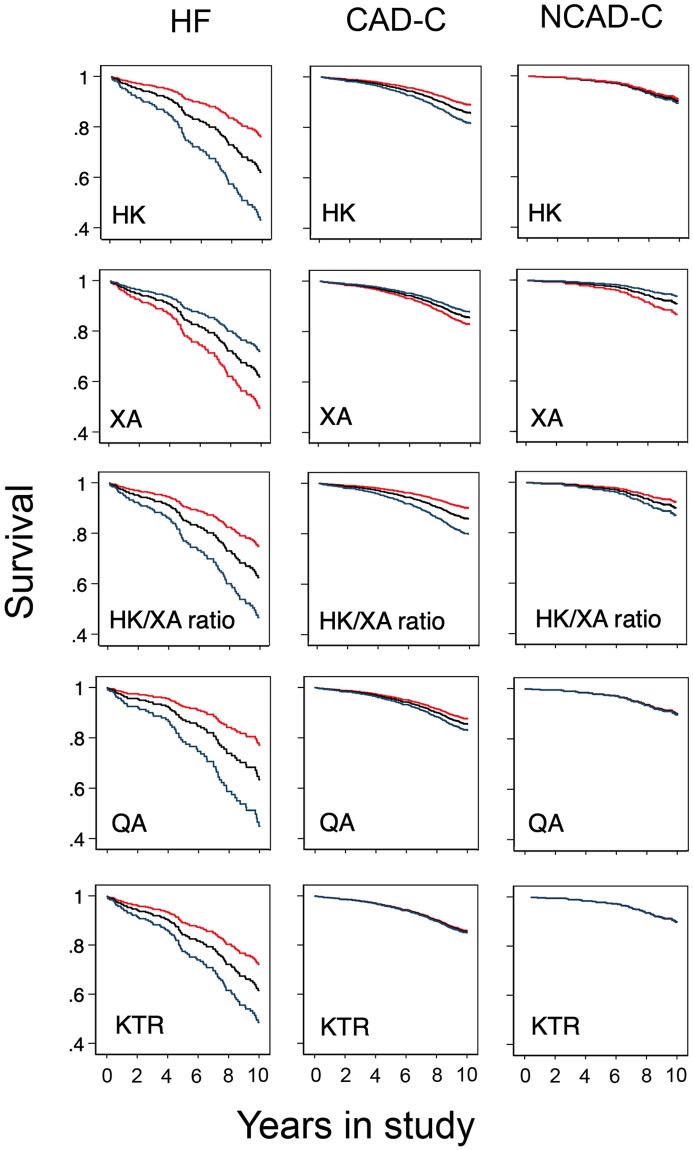
3-hydroxykynurenine, xanthurenic acid, quinolinic acid, the kynurenine-to-tryptophan ratio and survival. Predicted estimates from Cox regression with age, gender, current smoking, estimated glomerular filtration rate, diabetes mellitus, C-reactive protein and pyridoxal 5’phosphate as covariates. The analyses were performed in each group (HF, CAD-C and NCAD-C). The predicted estimates are shown at the mean level of each metabolite (black line), and 1 standard deviation above (red line) and below (blue line) the mean. Abbreviations: HF, heart failure; CAD-C, coronary artery disease controls; NCAD-C, non-coronary artery disease controls; HK, 3-hydroxykynurenic acid; XA, xanthurenic acid; KTR, kynurenine to tryptophan ratio; QA, quinolinic acid; SD, standard deviation.

In patients with heart failure, HK, QA, KTR and the HK/XA ratio were associated with increased risk of all-cause mortality, whereas XA was associated with a lower risk of all-cause mortality in all three groups. HK and the HK/XA ratio had a weaker association with mortality in the CAD-C group. Kyn, QA, and KTR were not associated with mortality in the control groups (Table 3). Fig 3 illustrate the strongest associations identified in adjusted analyses using Cox regression. Of note, the co-factor PLP was not associated with mortality (HR 0.90–1.06, p 0.209–0.706) in either group.

**Table 3:** Tryptophan, kynurenines and mortality in cases and controls ^a^.

	Heart Failure			NCAD-C		CAD-C	
	HR (95% CI)	p	Q ^b^	HR (95% CI)	p	HR (95% CI)	p
Trp	0.83 (0.7–1.0)	.10	.17	0.90 (0.7–1.2)	.45	1.07 (0.9–1.3)	.37
Kyn	1.41 (1.1–1.9)	.029*	.051	0.99 (0.7–1.46)	.98	0.99 (0.8–1.2)	.98
HK	1.77 (1.2–2.6)	.005*	.014*	0.91 (0.6–1.4)	.68	1.31 (1.1–1.6)	.004*
KA	0.90 (0.6–1.3)	.58	.62	0.79 (0.6–1.1)	.21	0.87 (0.7–1.0)	.12
XA	0.68 (0.7–1.2)	.011*	.025*	0.67 (0.5–0.9)	.029*	0.83 (0.7–0.9)	.037*
AA	1.07 (0.8–1.4)	.58	.61	1.04 (0.8–1.4)	.78	1.00 (0.9–1.2)	.99
HAA	1.14 (0.9–1.5)	.28	.39	0.73 (0.5–1.0)	.070	1.13 (1.0–1.3)	.14
QA	1.80 (1.1–2.9)	.013*	.028*	1.03 (0.8–1.4)	.86	1.18 (1.0–1.4)	.077
KTR	1.55 (1.1–2.2)	.009*	.023*	1.01 (0.7–1.5)	.96	0.98 (0.8–1.8)	.85
HK/XA	1.67 (1.3–2.2)	< .001**	.003*	1.31 (0.9–2.0)	.19	1.40 (1.2–1.7)	< .001**

Risk factors for all-cause mortality assessed in patients with heart failure, and in controls with (CAD-C) and without (NCAD-C) coronary heart disease. Each metabolite in separate model.

Abbreviations: AA, anthranilic acid; CI, confidence interval; HAA, 3-hydroxyanthranilic acid; HK, 3-hydroxykynurenine; HK/XA, 3-hydroxykynurenine-xanthurenic acid ratio; HR, hazard ratio; KA, kynurenic acid; KTR, kynurenine-tryptophan ratio; Kyn, kynurenine; p, p-value; SD; standard deviation; Trp, tryptophan; Q, q-value, QA, quinolinic acid; XA, xanthurenic acid.

^a^ Hazard ratio per 1 SD of the predictor by Cox proportional hazard analysis with all-cause mortality as the outcome, adjusted for age, gender, diabetes, current smoking, ejection fraction, estimated glomerular filtration rate, C-reactive protein, pyridoxal 5’phosphate and Trp (all metabolites in separate models. KTR not adjusted for Trp).

^b^ p-value adjusted for multiple comparisons in the study (Benjamini-Hochberg; 0.05)

* p-value or q-value < 0.05,

** p-value < 0.001

## Discussion

The mKP Kyn, HK, and QA and derived ratios KTR and HK/XA were higher in heart failure patients compared to controls with or without CAD. HK, QA, KTR and the HK/XA ratio were associated with higher all-cause mortality in heart failure. XA was consistently associated with lower mortality in all groups.

Strengths of this study include a systematic evaluation of CAD and EF with coronary angiography and ventriculography at baseline, an adequate sample size and comprehensive assessment of metabolites. The results were adjusted for relevant confounders and multiple comparisons. Patients in the control groups underwent elective coronary angiography, and thus might not be considered a healthy population. However, they had no history of, or received no treatment for heart failure. All matching procedures are associated with some risk of selection bias which cannot fully be addressed by increasing the number of matching variables, due to the risk of overmatching. Further, mortality in the control groups were considerably lower than in the heart failure group, reducing the statistical power. Finally, we determined biomarkers only at baseline. Despite good-to-fair intra-class correlation coefficients of the included biomarkers (0.67 to 0.44) over a period of 38 months [28], repeated measures would have been informative.

Kyn levels in our study were higher in heart failure compared to both control groups. This is in line with previous findings [10]. Adding to prior studies, we found higher HK and QA, and the ratios KTR and HK/XA were also elevated in patients with heart failure. Notably, the same findings were observed compared to controls with or without CAD. Despite the association between heart failure and CAD, our findings indicated that heart failure itself is associated with these alterations in mKP, independent of CAD.

The mKP as predictors of HF did not improve diagnostic prediction in a clinically meaningful way as evaluated by AUC derived from ROC curves, and sensitivity and specificity from logistic regression. This study did not include known predictors (biomarkers) of HF, like N-terminal prohormone of brain natriuretic peptide (NT-proBNP), and we could therefore not compare gold standard biomarkers with mKP, as predictors of HF. LVEF could have possibly been compared as a predictor of HF, but LVEF was included as a case definition parameter, generating spuriously high predictive value. However, as the KP is an essential and tightly regulated physiological pathway, it is unlikely that mKP concentrations will deviate so far from the norm that they can be used to clearly separate populations. As a further consequence of its key physiological role, it is likely to be affected by a range of human diseases. Even if mKP are likely poor diagnostic biomarkers, this does not preclude a role in the pathophysiology of heart failure.

Blood levels of pro-inflammatory mediators are elevated in heart failure [1]. This includes IFN-γ, an important inducer of IDO [5]. An *in vitro* study on monocytes exposed to IFN-γ identified that Kyn, KTR, HK, and QA were the most responsive to IFN-γ stimulation [29]. Furthermore, KTR is a marker of a cellular immune response, mainly reflecting monocyte activation [5]. The changes in circulating levels of mKP in heart failure observed in our data may thus reflect underlying monocyte activation, but might also related to reduced exercise in heart failure which can decrease clearance of mKP [30]. The changes in plasma KTR might also reflect increased TDO-activation and not only IDO-activation. Since this study does not include gene expression of TDO and IDO, dietary intake of Trp and hormone status, we cannot conclude that KTR represent only increased IDO-activation.

We observed associations between KTR, QA, and a trend for Kyn, and higher mortality in patients with heart failure. The control groups displayed attenuated, non-significant, effect sizes, perhaps indicating a vulnerability in patients with heart failure. Conceivably, myocardial fibrosis, a key driver of disease progression in heart failure, could be related to monocyte production of mKP. Such a hypothesis could be assessed in experimental studies [31–33]. Our findings could be related to underlying chronic inflammation induced by IFN-γ. However, IFN-γ has not emerged as a key prognostic predictor in heart failure, and studies show diverging results [7]. One possible explanation for this discrepancy might be that baseline measurements of Trp and Kyn are better long-term markers of inflammation than cytokines, as they are considered more stable in individuals over time [28, 34]. As IDO activation, and the concentrations of Kyn, and Trp are immune-regulatory, the relationship with inflammation might be complex [35].

The HK/XA ratio was most significantly associated with mortality. This reflects the significant associations between HK and increased mortality and XA and lower mortality, where XA is the immediate metabolic product of HK. The associations between high HK and HK/XA ratio and mortality were also observed in patients with CAD, but with lower effect sizes. Low XA was associated with higher mortality in all groups. A similar relationship between the HK/XA ratio and mortality has been described in renal transplant recipients [36], showing that the association is not limited to patients with heart failure. In experimental studies, HK accelerates apoptosis and endothelial dysfunction in mice [37], and apoptosis in human cells [38, 39]. Of the mKP, XA is likely the most potent antioxidant [40]. The HK/XA ratio may reflect an imbalance between apoptotic stimuli and antioxidant capacity. This ratio has been established as a functional marker of vitamin B6 status, and an elevated HK/XA ratio may reflect reduced activity of the PLP-dependent enzyme KAT [41]. However, the results in this study were not confounded by PLP [41] levels, and PLP levels were not associated with mortality. KAT enzymes generating HK from XA also depend on α-keto acids, such as α-ketoglutarate [42], which we did not measure, but are unlikely to be rate limiting. The biological significance of this ratio in heart failure warrants future investigation.

The potential for anti-inflammatory therapy in cardiovascular disease has been highlighted with the successful trial targeting the interleukin-1β pathway in patients with established CAD [43]. Experimental studies, starting with animal models, are needed to understand if manipulation of the KP can be of benefit to patients with heart failure. Limitations to this study, that should be taken into account in future studies, include lack of repeated measures of mKP, as well as hormone-, and dietary status. Immune profiling, including measurements of cytokines and chemokines that can influence the KP, would also be beneficial. This study is an observational study and we can thus only conclude with associations between HF and mKP and not causal mechanisms. However, our study identifying mKP as prognostic markers, may indicate a future therapeutic potential.

## Conclusion

In summary, we found that plasma levels of Kyn, HK, QA, and the ratios KTR and HK/XA are higher in heart failure compared to controls with or without CAD. Elevated plasma levels of HK and HK/XA were associated with increased mortality in both patients with heart failure and CAD, with larger effect size in patients with heart failure. QA and KTR were associated with increased mortality only in patients with heart failure. Low plasma levels of XA were associated with increased mortality in all groups. Our results indicate that future intervention on mKP may be of clinical interest.

## Supporting information

S1 Table Levels of tryptophan and kynurenines in cases and controls. Abbreviations: Trp, tryptophan; Kyn, kynurenine; HK, 3-hydroxykynurenine; KA, kynurenic acid; XA, xanthurenic acid; AA, anthranilic acid; HAA, 3-hydroxyanthranilic acid; QA, quinolinic acid; KTR, kynurenine-tryptophan ratio; HK/XA, 3-hydroxykynurenine-xanthurenic acid ratio. ^a^ Levels in median and (interquartile ranges). ^b^ Heart failure versus controls without coronary artery disease (NCAD-C, Mann-Whitney U test). ^c^ Heart failure versus controls with coronary artery disease (CAD-C, Mann-Whitney U test). * p-value < 0.05, ** p-value < 0.001.
(DOCX)

S1 Fig Receiver operating characteristic (ROC) curves, HF versus NCAD-C. ROC curves from unadjusted and adjusted multivariate logistic regression HF versus NCAD-C as outcome. Mod-1: regression with only the confounders diabetes, glomerular filtration rate, pyridoxal 5’phosphate and C-reactive protein included. Mod-2: mKP and ratios added to model. Abbreviations: AUC, area under curve; mKP, metabolites of the kynurenine pathway; Trp, tryptophan; Kyn, kynurenine; KTR, kynurenine-tryptophan ratio; HK/XA, 3-hydroxykynurenine-xanthurenic acid ratio.
(TIF)

S2 Fig Receiver operating characteristic (ROC) curves, HF versus CAD-C. ROC curves from unadjusted and adjusted multivariate logistic regression HF versus CAD-C as outcome. Mod-1: regression with only the confounders diabetes, glomerular filtration rate, pyridoxal 5’phosphate and C-reactive protein included. Mod-2: mKP and ratios added to model. Abbreviations: AUC, area under curve; mKP, metabolites of the kynurenine pathway; Trp, tryptophan; Kyn, kynurenine; HK, 3-hydroxykynurenine; KTR, kynurenine-tryptophan ratio; HK/XA, 3-hydroxykynurenine-xanthurenic acid ratio.
(TIF)
