# Correction: Plasma kynurenines and prognosis in patients with heart failure

**DOI:** 10.1371/journal.pone.0230056

**Published:** 2020-02-27

**Authors:** Anders Lund, Jan Erik Nordrehaug, Grete Slettom, Stein-Erik Hafstad Solvang, Eva Kristine Pedersen, Øivind Midttun, Arve Ulvik, Per Magne Ueland, Ottar Nygård, Lasse Melvaer Giil

The fifth author’s name is incorrect. The correct name is: Eva Kristine Pedersen.

In addition, the fourth, fifth, and last authors’ initials are indexed incorrectly in PubMed. The correct initials are: Solvang SH, Pedersen EK, and Giil LM.

As a result of the errors in the fourth and fifth authors’ names, these authors’ initials appear incorrectly in the citation. The correct citation is: Lund A, Nordrehaug JE, Slettom G, Solvang SH, Pedersen EK, Midttun Ø, et al. (2020) Plasma kynurenines and prognosis in patients with heart failure. PLoS ONE 15(1): e0227365. https://doi.org/10.1371/journal.pone.0227365
