# Supplementary material for: Plasma kynurenines and prognosis in patients with heart failure
Source: PLoS One. 2020 Jan 10;15(1):e0227365. doi: 10.1371/journal.pone.0227365 (PMC6953806; doi:10.1371/journal.pone.0227365)
Supplement: S1 Table — Abbreviations: Trp, tryptophan; Kyn, kynurenine; HK, 3-hydroxykynurenine; KA, kynurenic acid; XA, xanthurenic acid; AA, anthranilic acid; HAA, 3-hydroxyanthranilic acid; QA, quinolinic acid; KTR, kynurenine-tryptophan ratio; HK/XA, 3-hydroxykynurenine-xanthurenic acid ratio. a Levels in median and (interquartile ranges). b Heart failure versus controls without coronary artery disease (NCAD-C, Mann-Whitney U test). c Heart failure versus controls with coronary artery disease (CAD-C, Mann-Whitney U test). * p-value < 0.05, ** p-value < 0.001. (DOCX) [file pone.0227365.s001.docx]

**S1 Table. Levels of Tryptophan and Kynurenines in Cases and Controls.**

|  | **Heart Failure** |  | **Controls** | | | | | | |
| --- | --- | --- | --- | --- | --- | --- | --- | --- | --- |
|  |  |  | NCAD-C | | |  | CAD-C | | |
|  | Level ^a^ |  | Level ^a^ |  | p-value ^b^ |  | Level ^a^ |  | p-value ^c^ |
| Trp, μmol/L | 71.6 (22.0) |  | 72.5 (18.8) |  | .652 |  | 70.2 (18.8) |  | .240 |
| Kyn, μmol(L | 1.96 (0.85) |  | 1.62 (0.63) |  | <.001** |  | 1.68 (0.60) |  | <.001** |
| HK, nmol/L | 38.2 (29.6) |  | 30.7 (14.9) |  | <.001** |  | 30.7 (14.2) |  | <.001** |
| KA, nmol/L | 57.8 (36.0) |  | 45.9 (25.2) |  | <.001** |  | 47.7 (24.7) |  | <.001** |
| XA, nmol/L | 15.9 (13.3) |  | 14.9 (9.9) |  | .057 |  | 14.2 (9.7) |  | .005 |
| AA, nmol/L | 16.0 (7.6) |  | 14.2 (6.3) |  | <.001** |  | 14.3 (6.9) |  | <.001** |
| HAA, nmol/L | 35.9 (19.1) |  | 32.8 (17.4) |  | .003* |  | 33.6 (18.6) |  | .043* |
| QA, nmol/L | 476 (244) |  | 379 (160) |  | <.001** |  | 392.4 (44) |  | <.001** |
| KTR | 28.1 (13.8) |  | 23.2 (8.1) |  | <.001** |  | 24.0 (8.4) |  | <.001** |
| HX/XA | 2.45 (1.7) |  | 2.01 (1.0) |  | <.001** |  | 2.08 (1.3) |  | <.001** |
