# Supplementary figures and images for: Plasma kynurenines and prognosis in patients with heart failure
Source: PLoS One. 2020 Jan 10;15(1):e0227365. doi: 10.1371/journal.pone.0227365 (PMC6953806; doi:10.1371/journal.pone.0227365)

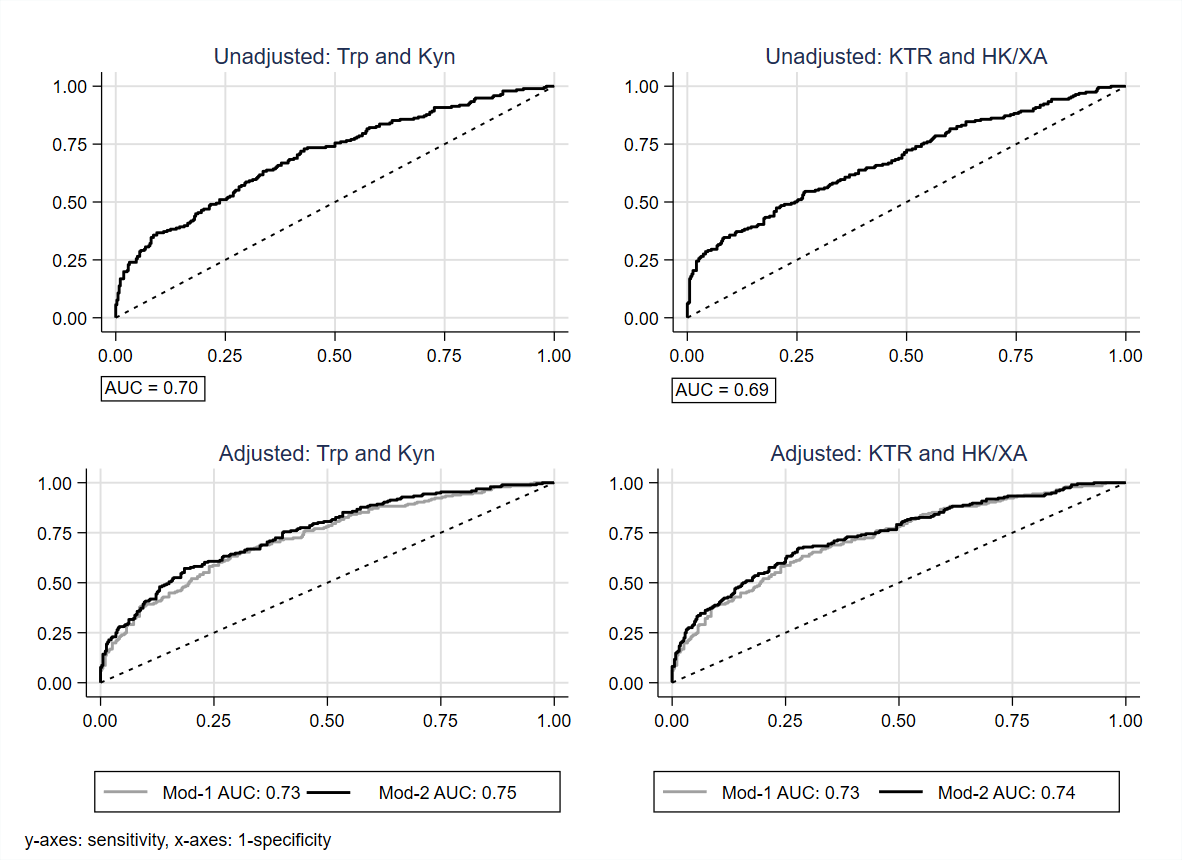

Supplement: S1 Fig — ROC curves from unadjusted and adjusted multivariate logistic regression HF versus NCAD-C as outcome. Mod-1: regression with only the confounders diabetes, glomerular filtration rate, pyridoxal 5’phosphate and C-reactive protein included. Mod-2: mKP and ratios added to model. Abbreviations: AUC, area under curve; mKP, metabolites of the kynurenine pathway; Trp, tryptophan; Kyn, kynurenine; KTR, kynurenine-tryptophan ratio; HK/XA, 3-hydroxykynurenine-xanthurenic acid ratio. (TIF) [file pone.0227365.s002.tif]

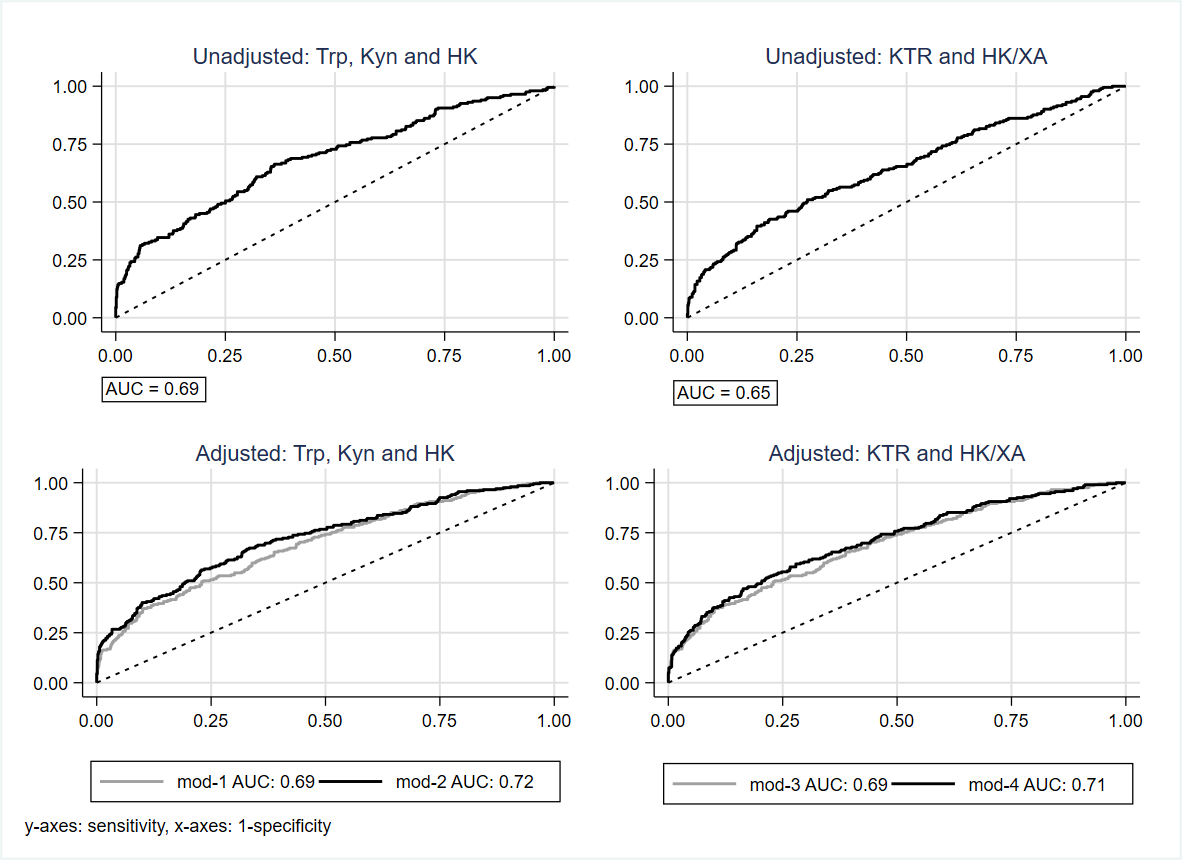

Supplement: S2 Fig — ROC curves from unadjusted and adjusted multivariate logistic regression HF versus CAD-C as outcome. Mod-1: regression with only the confounders diabetes, glomerular filtration rate, pyridoxal 5’phosphate and C-reactive protein included. Mod-2: mKP and ratios added to model. Abbreviations: AUC, area under curve; mKP, metabolites of the kynurenine pathway; Trp, tryptophan; Kyn, kynurenine; HK, 3-hydroxykynurenine; KTR, kynurenine-tryptophan ratio; HK/XA, 3-hydroxykynurenine-xanthurenic acid ratio. (TIF) [file pone.0227365.s003.tif]
